# Incommensurate atomic density waves in the high-pressure IVb phase of barium

**DOI:** 10.1107/S2052252517000264

**Published:** 2017-01-21

**Authors:** Alla Arakcheeva, Maxim Bykov, Elena Bykova, Leonid Dubrovinsky, Phil Pattison, Vladimir Dmitriev, Gervais Chapuis

**Affiliations:** a Phase Solutions Co Ltd, ch. des Mésanges 7, Lausanne 1012, Switzerland; b Laboratoire de Physique de la Matière Complexe, EPFL, Lausanne 1015, Switzerland; c Institute of Experimental Geochemistry and Geophysics (BGI), University of Bayreuth, Bayreuth 95440, Germany; d Laboratoire de Cristallographie, IPSB, EPFL, Lausanne 1015, Switzerland; e Swiss–Norwegian Beamlines, ESRF, avenue des Martyrs 71, Grenoble 38000, France

**Keywords:** high-pressure barium phases, incommensurately modulated structures, Ba IVb, atomic density waves, host–guest structures

## Abstract

The host–guest structures of elements at high pressure discovered a decade ago still leave many open questions due to the lack of precise models based on full exploitation of the diffraction data. This concerns in particular Ba IV, which is stable in the range 12–45 GPa. With the example of phase Ba IVb, which is characterized here for the first time, a systematic analysis is presented of possible host–guest structure models based on high-quality single-crystal diffraction data obtained with synchrotron radiation at six different pressures between 16.5 and 19.6 GPa. It is shown that a new incommensurately modulated (IM) structure model better fits the experimental data. Unlike the composite models which are commonly reported for the Ba IV phases, the IM model reveals a density wave and its pressure-dependent evolution. The crucial role played by the selected model in the interpretation of structure evolution under pressure is discussed. The findings give a new experimental basis for a better understanding of the nature of host–guest structures.

## Abstract 2

Using single-crystal synchrotron radiation X-ray experiments, the host–guest structure of the Ba IVb high-pressure phase has been solved for the first time. The proposed systematic analysis of possible host–guest structure models shows that the structure belongs to the incommensurately modulated type. This model reveals an atomic density wave and its pressure-dependent evolution between 16.5 and 19.6 GPa.

## 1. Introduction   

In the last decade, important developments in single-crystal high-pressure X-ray diffraction experiments (Merlini & Hanfland, 2013 ▸; Kantor *et al.*, 2012 ▸; McMahon *et al.*, 2013 ▸) led to the discovery of completely new characteristics in the phase diagrams of the elements. These developments have shown that a surprising degree of complexity exists in the structures of some elements and in their physical behaviour at high pressure (McMahon & Nelmes, 2006 ▸; Degtyareva, 2010 ▸; Fabbris *et al.*, 2015 ▸). This provides a rich testing ground for fundamental physics, chemistry and material sciences.

Many recently reported crystal structures of elements at high pressure (McMahon & Nelmes, 2006 ▸; Degtyareva, 2010 ▸) are not only very complex but, in addition, often lose their three-dimensional periodicity (Loa *et al.*, 2012 ▸; Nelmes *et al.*, 1999 ▸; McMahon & Nelmes, 2004*a* ▸; McMahon *et al.*, 2000 ▸, 2006 ▸, 2007 ▸; McMahon, Rekhi & Nelmes, 2001 ▸; Lundegaard *et al.*, 2009 ▸; Degtyareva, McMahon & Nelmes, 2004*a* ▸; Schwarz *et al.*, 2003 ▸). This means that any structural description using the classical approach can result in crude and inappropriate approximations (Loa *et al.*, 2012 ▸; Nelmes *et al.*, 2002 ▸; McMahon, Nelmes & Rekhi, 2001 ▸; Degtyareva, McMahon *et al.*, 2004 ▸). The real challenge is to recover the correct symmetrical properties hidden behind the complexity of the diffraction patterns, in order to reveal their detailed and true atomic arrangement (McMahon *et al.*, 2007 ▸; Schwarz *et al.*, 2003 ▸; Perez-Mato *et al.*, 2006 ▸).

Barium is an interesting and very typical case of structural complexity. It exhibits a pressure-dependent structure evolution from a body-centred cubic (b.c.c.) phase (Ba I at *P* < 5.5 GPa), followed by a hexagonally close-packed (h.c.p.) phase (Ba II at 5.5 < *P* < 12 GPa), followed by the very complex so-called host–guest (H-G) structure of Ba IV (Loa *et al.*, 2012 ▸; Nelmes *et al.*, 1999 ▸; McMahon & Nelmes, 2004*a* ▸), which is an equilibrium phase stable between 12 and 45 GPa at room temperature (Loa *et al.*, 2012 ▸; Nelmes *et al.*, 1999 ▸; McMahon & Nelmes, 2004*b* ▸; Kenichi, 1994 ▸). The H-G structure has been described as an incommensurate composite model with two interpenetrating H and G substructures, similar to many other elements at high pressure (Nelmes *et al.*, 1999 ▸; McMahon & Nelmes, 2004*a* ▸; McMahon *et al.*, 2000 ▸, 2006 ▸, 2007 ▸; McMahon, Rekhi & Nelmes, 2001 ▸; Lundegaard *et al.*, 2009 ▸; Degtyareva, McMahon & Nelmes, 2004*a* ▸; Schwarz *et al.*, 2003 ▸). There is a clear differentiation between atoms forming the H framework and the G atoms forming chains located in parallel channels.

Unlike other elements, Ba IV exhibits extremely elaborate pressure-dependent transformations of the H-G structure. With increasing pressure, phases Ba IVa, Ba IVb and Ba IVc have been identified (Loa *et al.*, 2012 ▸; Nelmes *et al.*, 1999 ▸; McMahon & Nelmes, 2004*a* ▸). Precise structural characterizations of these phases are missing or incomplete. The Ba IVa structure has been solved by considering separately the H and G partial structures using a three-dimensional model for each of them (Nelmes *et al.*, 1999 ▸; McMahon & Nelmes, 2004*a* ▸). For the most complex phase of Ba IVc, the authors proposed a three-dimensional periodic model including 768 atoms in a supercell approximation with 72 times the volume of the basic host unit cell including 99 independent Ba atoms (Loa *et al.*, 2012 ▸). In principle, this implies the existence of an equal number of different electronic states, which simply looks unrealistic. Resolving the precise structures of these barium phases is therefore a key point in order to understand better the behaviour of metals under high pressure.

The degree of complexity of Ba IVc (Loa *et al.*, 2012 ▸) is not comparable with the proposed H-G model of Ba IVa (Nelmes *et al.*, 1999 ▸; McMahon & Nelmes, 2004*a* ▸), with two small subcells and only two independent atoms. While the strategy of adopting a three-dimensional superstructure model to approximate the effects of modulation and the presence of satellite reflections is commonplace, the above example demonstrates that the structural complexity can quickly become unmanageable.

From previous studies of Ba high-pressure (HP) phases, it appears that no crystallographic data are available for Ba IVb and its structure is still unresolved. In order to shed light on the unknown HP phase Ba IVb, we collected a sequence of single-crystal diffraction intensities at room temperature using synchrotron radiation in the pressure range between 16.5 and 21.8 GPa, where Ba IVb is stable. For each pressure, all reflections were indexed in terms of a single incommensurately modulated structure, thus leading to a structure solution within the (3+1)-dimensional superspace symmetry approach. [In the following, the terminology described by Janssen *et al.* (2007 ▸) will be used.] With the example of Ba IVb, we show that a systematic analysis of the indexed reflections allows different models for the H-G structures. For the first time we consider the incommensurately modulated (IM) model along with the incommensurate composite, which is usually applied for H-G structures (Nelmes *et al.*, 1999 ▸; McMahon & Nelmes, 2004*a* ▸; McMahon *et al.*, 2000 ▸, 2006 ▸, 2007 ▸; McMahon, Rekhi & Nelmes, 2001 ▸; Lundegaard *et al.*, 2009 ▸; Degtyareva, McMahon & Nelmes, 2004*a* ▸; Schwarz *et al.*, 2003 ▸). Tests of the models clearly favour the IM one for Ba IVb. The IM structure of Ba IVb reveals a density wave in the channel atoms (G substructure) which is pressure-dependent. This wave is created by the shift of atomic positions in the crystal bulk, which evolve towards a uniform distribution at higher pressure. For comparison, this wave is absent in the similar structure of Rb IV (McMahon, Rekhi & Nelmes, 2001 ▸), which was solved as a composite with periodic H and G sub­structures. The IM model exhibits the unique property that it can generate a unified and consistent series of structural models of the same or other compounds at different pressures, and hence can provide a tool for structure prediction (Arakcheeva & Chapuis, 2008 ▸).

## 2. Experimental   

### 2.1. Sample preparation   

Small pieces of barium were cut from an ingot of high purity (99.99%; Sigma–Aldrich) and loaded into a BX90 type diamond anvil cell (DAC) equipped with Boehler–Almax diamonds (500 µm culettes) (Kantor *et al.*, 2012 ▸). A rhenium gasket indented to about 40 µm with a hole 200 µm in diameter served as the pressure chamber. Neon was used as the pressure-transmitting medium. In order to avoid contamination of the sample, all manipulations during sample loading were performed under an inert argon atmosphere. A small ruby chip, used as a pressure standard, was enclosed with the sample in the DAC. The sample was pressurized to 15 GPa at room temperature and subsequently annealed at 200°C for 12 h. After slow cooling to room temperature, single crystals of phase Ba IVb were obtained.

### 2.2. X-ray diffraction   

Diffraction intensities were collected at various pressures between 15 and 27 GPa at room temperature using a wavelength of 0.4151 Å on the ESRF beamline ID09A. The collected ω-scan technique was used, with Δω = 0.5° and an exposure time of 1 s. The general beamline setup is described by Merlini & Hanfland (2013 ▸). Data processing (peak intensity integration, background evaluation, lattice parameters, frame scaling and reciprocal-space reconstructions) was performed with the *CrysAlis PRO* software (Rigaku Oxford Diffraction, 2014 ▸). Reflections with *I* < 2σ(*I*) were excluded from further consideration. Reflections with *I* > 2σ(*I*) were averaged according to symmetry. Structural analysis and all calculations were performed using the *JANA2006* software system (Petříček *et al.*, 2014 ▸).

## 3. Results   

In our experiments, two different diffraction ranges can be distinguished between 16.5 (1) and 21.8 (1) GPa. The phase described by Loa *et al.* (2012 ▸) as Ba IVc is observed above 20 GPa together with Ba IVb, which is a single phase below this pressure. Disparity with the pressure range of 18–21 GPa previously published for Ba IVc is almost certainly due to the neon gas pressure-transmitting medium used for the first time in the present Ba IV study.

Structure solutions of Ba IVb at 16.5 (1), 17.4 (1), 18.2 (1), 18.5 (1), 19.0 (1) and 19.6 (1) GPa are reported and discussed below.

### 3.1. Reciprocal space and superspace symmetry   

**Figure 1:**
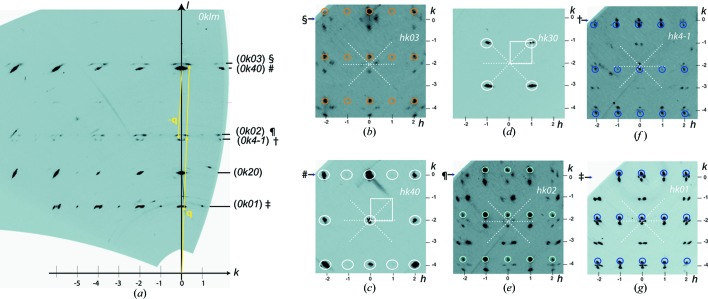
Reciprocal-space sections measured at 19.6 (1) GPa containing (*a*) 0*klm* and (*b*)–(*g*) *hklm* reflections. White, dark-blue, turquoise and orange circles indicate the main reflections and first-, second- and third-order satellites, respectively. The special characters in part (*a*) refer to the corresponding lines in the other panels. All reflections are indexed by the reciprocal-lattice vectors **H** = *h* a* + *k* b* + *l* c* + *m* **q**, with **q** = βb* + γc* [yellow arrows in part (*a*)]. In parts (*c*) and (*d*), the a*b* reciprocal lattice plane (white square) is defined by the strong main reflections *hk*30 and *hk*40. In parts (*b*), (*e*), (*f*) and (*g*), the satellites indicated by circles belong to independent reflections related to others by the (110) and (010) twinning planes (dashed white lines).

The complete interpretation of the diffracted intensities is illustrated in Fig. 1 ▸ with the diffraction measurements obtained at 19.6 (1) GPa. The whole set of reflections was indexed as *hklm* with the wavevector **H** = *h* a*+ *k* b* + *l* c* + *m* **q** using lattice parameters *a* ≃ *b* ≃ 11.5 Å and *c* ≃ 4.6 Å and the incommensurately modulated vector **q** = βb* + γc* ≃ 0.1b* + 1.36c*. The strong *hkl*0 reflections are considered the main ones; all others *hklm* (*m* ≠ 0) are satellites. The index *m* = 1, 2 and 3 corresponds to three orders of satellites observed by diffraction (Fig. 1 ▸).

**Figure 2:**
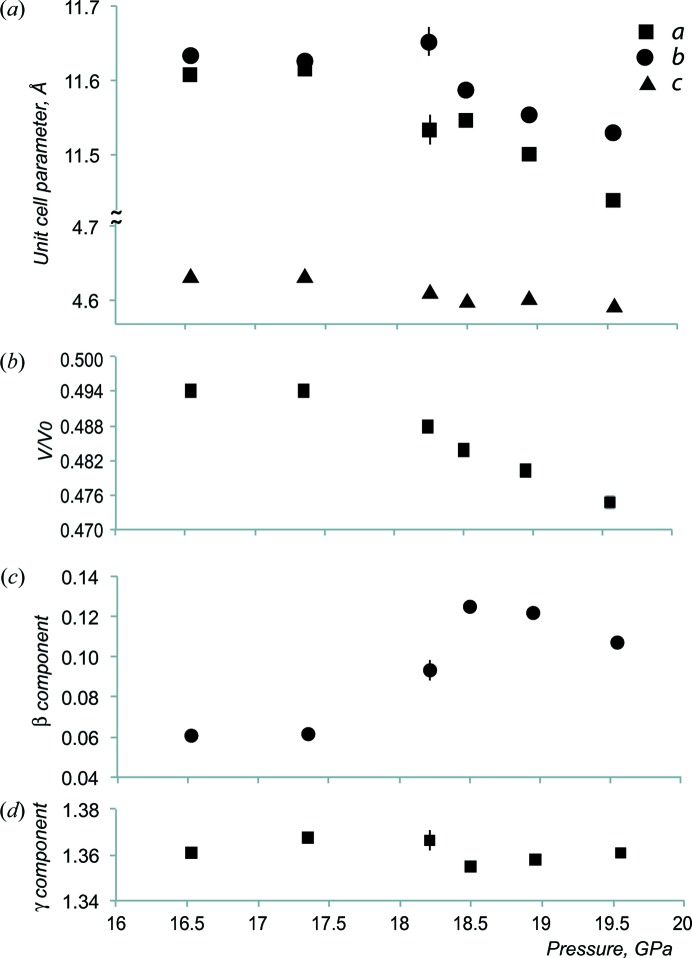
Pressure dependence of the unit-cell parameters and components β and γ of the modulation vector **q** = βb* + γc*. (*a*) The *a* and *b* parameters are sensitive to pressure, while *c* is rather stable, *c* = 4.61 ± 0.02 Å. (*b*) The unit-cell volume ratio relative to the phase at ambient temperature and normal pressure. (*c*) The β component of the vector **q** changes from 0.06 ± 0.002 to 0.115 ± 0.010 between 17.4 (1) and 18.5 (1) GPa. (*d*) The γ component is stable, with a value equal to 1.36 ± 0.005.

Despite the tetragonal character (Figs. 1 ▸ *c* and 1 ▸ *d*) of the main reflections, the refined lattice parameters point to a lower symmetry (Fig. 2 ▸ *a*). For all six measurements, averaging of the reflection intensities shows unacceptable reliabilities of *R*_int_ ≃ 0.55 for each group of symmetry-equivalent reflections. An improved reliability with *R*_int_ = 0.04–0.14 was obtained in the monoclinic system with the unique axis *a*. The pseudo-tetragonal appearance of the reciprocal-space patterns (Fig. 1 ▸) can be understood as a consequence of twins linked by the (010), 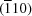 and (110) mirror planes inherited from the tetragonal Ba IVa modification stable at lower pressure (Nelmes *et al.*, 1999 ▸; McMahon & Nelmes, 2004*a* ▸).

Using the refined lattice parameters and components of the modulation vector **q** (Figs. 2 ▸ *a*, 2 ▸ *c* and 2 ▸ *d*), the integration of experimental intensities was performed for the six diffraction data sets. For each pressure, about 18 000 reflections were used for the structure solution and refinement, of which 600–700 had *I* > 2σ(*I*).

The (3+1)-dimensional monoclinic superspace group *P*2_1_/*b*(0βγ)00 was derived from the observed systematic extinctions.

### 3.2. Incommensurately modulated structure *versus* composite model   

Indexing of the diffraction reflections and the derived monoclinic superspace group are consistent with two different structure models: incommensurately modulated (IM) and modulated composite (COMP). In both models, two independent atomic sites describe the framework atoms, Ba_fr_ (called ‘host’, H, in COMP), and one site defines the channel atoms, Ba_ch_, (called ‘guest’, G, in COMP). The principal distinction between IM and COMP concerns the *c* lattice parameter, which is different for H (*c*_H_ ≃ 4.6 Å) and G (*c*_G_ ≃ 3.4 Å) in COMP, while it is unique (*c* ≃ 4.6 Å) in IM. All details of the differences between the IM and COMP models are given in the supporting information. We have tested and refined both models for Ba IVb (supporting information, Tables S2–S7). The validity of and preference for IM are confirmed by the low reliability indices *R* (Fig. S1), which were calculated for both COMP and IM for the six different pressures. For IM, *R*_main_ = 0.043–0.056 and *R*_all_ = 0.091–0.099 for the main and all reflections, respectively, while essentially higher *R*_main_ = 0.095–0.12 and *R*_all_ = 0.139–0.17 were obtained for COMP.

### 3.3. The density wave and its pressure-sensitive evolution   

**Figure 3:**
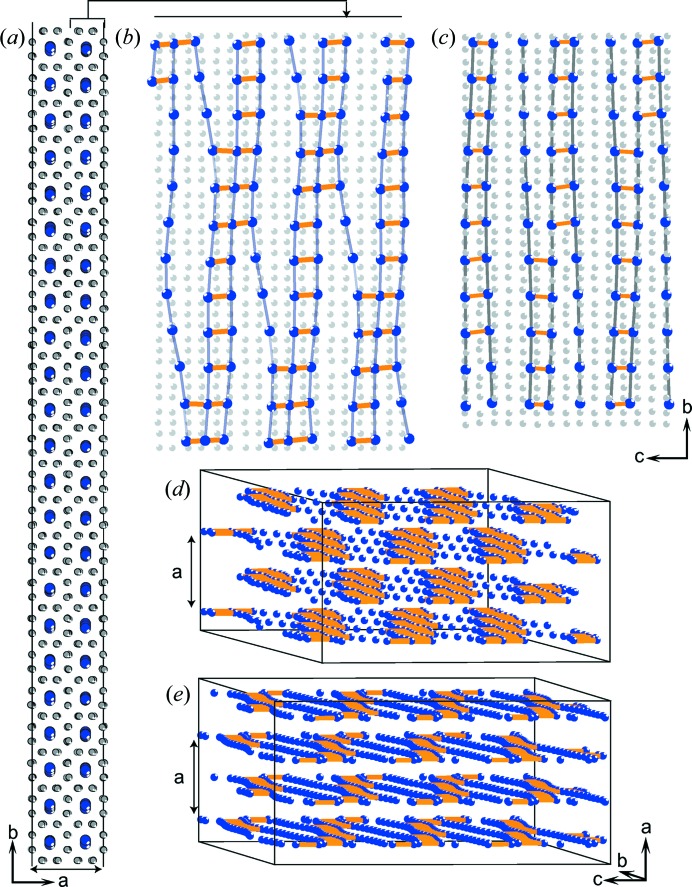
The incommensurately modulated structure Ba IVb. Both Ba_fr_ (grey) and Ba_ch_ (blue) atoms are subject to positional modulation. The structure is periodic along the *a* axis and aperiodic in the *bc* planes. (*a*) A portion of the structure given in the *ab* projection, illustrating the weak modulation of all the atoms in the *a* and *b* directions. (*b*) and (*c*) The *bc* projection of a layer of thickness 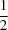 *a* at (*b*) 19.6 (1) and (*c*) 16.5 (1) GPa. The projection shows that the main structure modulation concerns the displacements of Ba_ch_ atoms along the *c* axis, *i.e.* along the channels formed by Ba_fr_ atoms. The displacements induce variation in the Ba_ch_—Ba_ch_ distances. The orange sticks link atoms distant by 2.90 to 3.8 Å, showing a wave of dense and sparse arrangements of Ba_ch_ atoms. (*d*) and (*e*) The alternating distribution of the layers shown along the *a* axis at (*d*) 19.6 and (*e*) 16.5 GPa. Ba_fr_ atoms have been omitted to highlight the chessboard-like distribution of dense and sparse regions.

**Figure 4:**
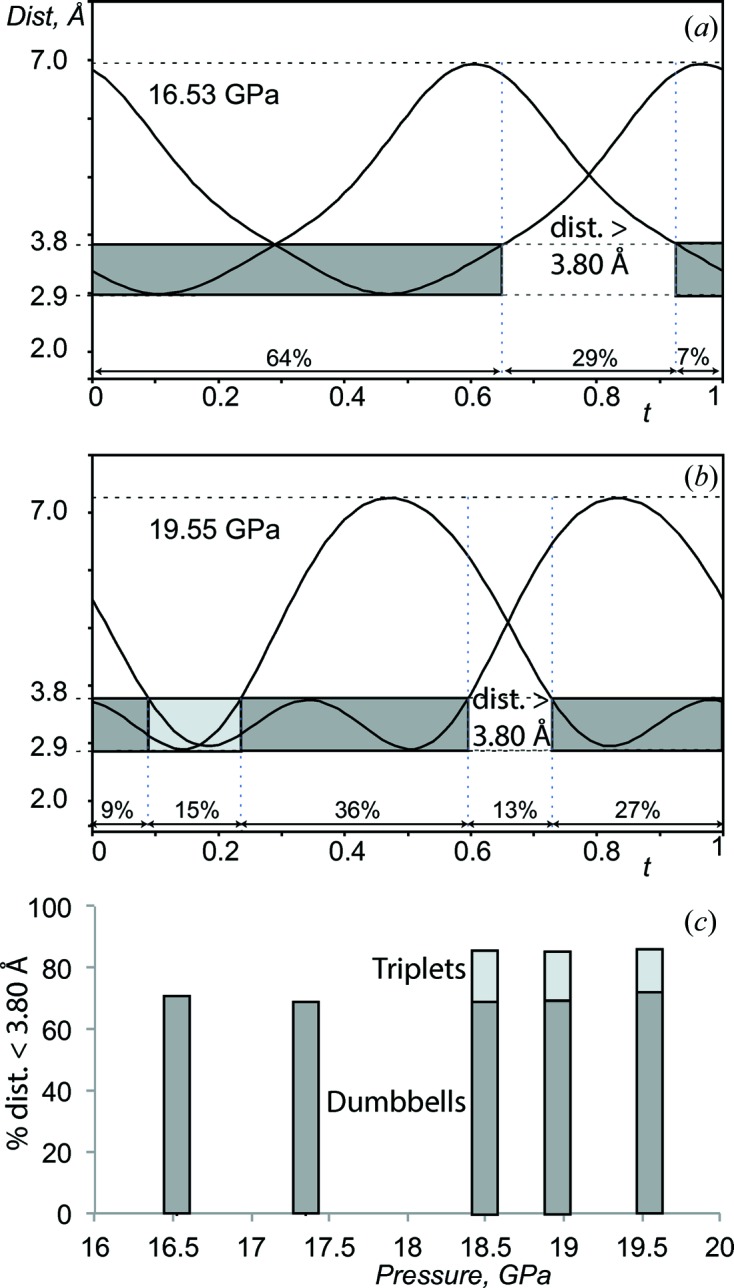
Statistical analysis of the Ba_ch_—Ba_ch_ distances in the channels. (*a*) and (*b*) *t*-Plots of the closest Ba_ch_—Ba_ch_ distances and their statistical distribution at (*a*) 16.5 and (*b*) 19.6 GPa. Each solid line corresponds to a distance varying between 2.9 and about 7 Å. The domain intervals indicate one (dark grey) and two (light grey) interatomic contacts associated with the formation of dumb-bells and triplets, respectively, in the 2.9–3.8 Å range. (*c*) The pressure dependence of the dumb-bell and triplet content.

The IM model reveals an essential and specific feature of the Ba IVb phase, namely the presence of a density wave in the channels of the structure (Fig. 3 ▸). This wave arises from the nearest Ba_ch_—Ba_ch_ distance variation in the channels (Fig. 4 ▸, and Section S3 in the supporting information). 70–85% of these distances are in the range 2.9–3.8 Å (Figs. 4 ▸ *a* and 4 ▸ *b*), forming dumb-bells and triplets along the channel (Figs. 4 ▸ *b* and 4 ▸ *c*). This results in dense and sparse arrangements of the Ba_ch_ atoms, forming a wave in the *bc* layers (Figs. 3 ▸ *b* and 3 ▸ *c*). This wave changes with pressure, increasing from 16.5 to 19.6 GPa towards the formation of a more uniform chessboard-like distribution of the dense and sparse regions in the bulk of the crystal (Figs. 3 ▸ *d* and 3 ▸ *e*). The pressure dependence of the unit-cell parameters (Figs. 2 ▸ *a* and 2 ▸ *b*), and especially of the β component of the modulation vector (Fig. 2 ▸ *c*), confirms this pressure-sensitive structural evolution appearing between 17.4 (1) and 18.2 (1) GPa.

## 4. Discussion   

A key point of our new results concerns the measurement, interpretation and indexing of *hklm* reflections with *l* ≠ 0 and *m* ≠ 0 (for instance, 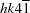 in Fig. 1 ▸). Despite having much weaker intensity than the strong *hkl*0 main reflections, this group concerns up to 30–50% of the measured reflections in our experiments (Fig. S1 in the supporting information). The existence of these reflections was mentioned previously for Ba IV (McMahon *et al.*, 2007 ▸), but without any further consideration. By ignoring them, it is thus possible to interpret the measurements in terms of two independent interpenetrating H and G substructures, which are both periodic with common lattice parameters *a* and *b* but with different *c*: *c*_H_ ≠ *c*_G_, the aperiodicity originating from the *c*_H_/*c*_G_ ratio. Such typical non-modulated H-G structures have been reported for many elements, such as Sr V (McMahon *et al.*, 2007 ▸), Rb IV (McMahon, Rekhi & Nelmes, 2001 ▸), K III (McMahon *et al.*, 2006 ▸), tI19 Na (Lundegaard *et al.*, 2009 ▸), Sb II, As III and Bi III (Degtyareva, McMahon & Nelmes, 2004*a* ▸), and also Ba IVa (Nelmes *et al.*, 1999 ▸; McMahon & Nelmes, 2004*a* ▸). The observation of the above-mentioned reflections clearly points to modulations in any H-G structure. By improving the X-ray diffraction experiment, these reflections have been included in the refinement, and the structure models of Bi III (McMahon *et al.*, 2007 ▸) and Sb II (McMahon *et al.*, 2007 ▸; Schwarz *et al.*, 2003 ▸) were re-interpreted in terms of modulated composites (COMP) with variation in the interatomic distances in both H and G and also between them. No attempt to apply an IM model has been done before for any H-G structure. In the present study, we show that, with the example of Ba IVb, this attempt not only makes sense, but it favours the IM model.

Similar to the reports on Bi III (McMahon *et al.*, 2007 ▸) and Sb II (McMahon *et al.*, 2007 ▸; Schwarz *et al.*, 2003 ▸), we consider the strong *hkl*0 reflections as the main ones. However, all other reflections, *i. e. hklm* (*m* ≠ 0), are interpreted as satellites in our model. The difference concerns the *hk*0*m* (*m* ≠ 0) reflections, which are considered as main reflections for the G substructure in COMP. The reported scheme of the reflection distribution in Ba IVa (Nelmes *et al.*, 1999 ▸) is very similar to that found here for Ba IVb. This is a strong hint to test the Ba IVa–Ba IVb transformation within the framework of an identical IM model, applying variation of the incommensurability vector **q** = βb* + γc*. It would be challenging to test the IM model for the other host–guest structures of the elements at different pressures, for instance the incommensurate-to-incommensurate phase transition in antimony (Degtyareva, McMahon & Nelmes, 2004*b* ▸).

With improved diffraction measurements, the IM model gives the possibility of testing whether a similar or different density wave from Ba IVb is present in other H-G structures of the elements. Such a wave cannot be detected using a non-modulated composite model arising from an incomplete set of weak experimental reflections. The reason is that such a model *a priori* postulates the periodicity of the G atoms. However, atomic groupings, similar to the dumb-bells and triplets in Ba IVb, were found in the G chains of Bi III and Sb II using the weak *hklm* (*l* ≠ 0 and *m* ≠ 0) reflections for the COMP model (McMahon *et al.*, 2007 ▸; Schwarz *et al.*, 2003 ▸). It is not possible to predict which structure model, COMP or IM, is more relevant for the characterization of an H-G structure of an element at various pressures and temperatures. Only a complete structure refinement can show which model better fits the experimental diffraction intensities.

## 5. Conclusions   

The interpretation of an intriguing high-pressure induced phenomenon is extremely sensitive to the structural model used. This is the so-called ‘one-dimensional chain melting’, which has recently been reported for K (McBride *et al.*, 2015 ▸) and Rb (McMahon & Nelmes, 2004*a* ▸) H-G structures. The main experimental observation is a decrease and vanishing of intensity for *hk*0*m* reflections under certain pressure/temperature conditions (McBride *et al.*, 2015 ▸; McMahon & Nelmes, 2004*a* ▸). Assuming the composite structure models, this phenomenon is interpreted as the successive dis­appearance of interactions between H and G and even between G atoms, the so-called ‘melting’ of G chains inside a crystal formed of H atoms (McBride *et al.*, 2015 ▸; McMahon & Nelmes, 2004*a* ▸). By assuming an IM structure model, this phenomenon can be interpreted as the disappearance of satellite reflections. The crystal structure becomes periodic if the intensity of the satellites is equal to zero. Unlike in the COMP models, this means that the structure recovers a new equilibrium, with periodic interactions in both H and G and between them. It thus appears that the conclusions are completely opposite to each other depending on the structure model. Hence, high-quality single-crystal data collection, accurate and detailed analysis of the diffraction data and reliable structure refinements are essential to improve our knowledge of the high-pressure H-G structures found for the elements.

Our results obtained for Ba IVb open some new perspectives for studying the complexity of the elements under high pressure.

## Supplementary Material


13062EqEcIW

Crystal structure: contains datablock(s) global, I, II, III, IV, V, VI. DOI: 10.1107/S2052252517000264/yu5011sup1.cif

Structure factors: contains datablock(s) I. DOI: 10.1107/S2052252517000264/yu5011Isup2.hkl

Structure factors: contains datablock(s) II. DOI: 10.1107/S2052252517000264/yu5011IIsup3.hkl

Structure factors: contains datablock(s) III. DOI: 10.1107/S2052252517000264/yu5011IIIsup4.hkl

Structure factors: contains datablock(s) IV. DOI: 10.1107/S2052252517000264/yu5011IVsup5.hkl

Structure factors: contains datablock(s) V. DOI: 10.1107/S2052252517000264/yu5011Vsup6.hkl

Structure factors: contains datablock(s) VI. DOI: 10.1107/S2052252517000264/yu5011VIsup7.hkl

Additional information, figure and tables. DOI: 10.1107/S2052252517000264/yu5011sup8.pdf

## References

[bb2] Arakcheeva, A. & Chapuis, G. (2008). *Acta Cryst.* B**64**, 12–25. 10.1107/S010876810705923X 18204207

[bb3] Degtyareva, O. (2010). *High. Press. Res.* **30**, 343–371.

[bb4] Degtyareva, O., McMahon, M. I., Allan, D. R. & Nelmes, R. J. (2004). *Phys. Rev. Lett.* **93**, 205502. 10.1103/PhysRevLett.93.205502 15600936

[bb5] Degtyareva, O., McMahon, M. I. & Nelmes, R. J. (2004*a*). *High. Press. Res.* **24**, 319–356.

[bb6] Degtyareva, O., McMahon, M. I. & Nelmes, R. J. (2004*b*). *Phys. Rev. B*, **70**, 184119.

[bb8] Fabbris, G., Lim, J., Veiga, L. S. I., Haskel, D. & Schilling, J. S. (2015). *Phys. Rev. B*, **91**, 085111.

[bb9] Janssen, T., Chapuis, G. & de Boissieu, M. (2007). *Aperiodic Crystals: From Modulated Phases to Quasicrystals.* Oxford University Press.

[bb7] Kantor, I., Prakapenka, V., Kantor, A., Dera, P., Kurnosov, A., Sinogeikin, S., Dubrovinskaia, N. & Dubrovinsky, L. (2012). *Rev. Sci. Instrum.* **83**, 125102. 10.1063/1.4768541 23278021

[bb11] Kenichi, T. (1994). *Phys. Rev. B*, **50**, 16238–16246. 10.1103/physrevb.50.16238 9976008

[bb12] Loa, I., Nelmes, R. J., Lundegaard, L. F. & McMahon, M. I. (2012). *Nat. Mater.* **11**, 627–632. 10.1038/nmat3342 22683822

[bb13] Lundegaard, L. F., Gregoryanz, E., McMahon, M. I., Guillaume, C., Loa, I. & Nelmes, R. J. (2009). *Phys. Rev. B*, **79**, 064105.

[bb14] McBride, E. E., Munro, K. A., Stinton, G. W., Husband, R. J., Briggs, R., Liermann, H.-P. & McMahon, M. I. (2015). *Phys. Rev. B*, **91**, 144111.

[bb15] McMahon, M. I., Bovornratanaraks, T., Allan, D. R., Belmonte, S. A. & Nelmes, R. J. (2000). *Phys. Rev. B*, **61**, 3135–3138.

[bb16] McMahon, M. I., Degtyareva, O., Nelmes, R. J., van Smaalen, S. & Palatinus, L. (2007). *Phys. Rev. B*, **75**, 184114.

[bb17] McMahon, M. I., Loa, I., Stinton, G. W. & Lundegaard, L. F. (2013). *High. Press. Res.* **33**, 485–500.

[bb18] McMahon, M. & Nelmes, R. (2004*a*). *Z. Kristallogr.* **219**, 742–748.

[bb19] McMahon, M. I. & Nelmes, R. J. (2004*b*). *Phys. Rev. Lett.* **93**, 055501. 10.1103/PhysRevLett.93.055501 15323704

[bb20] McMahon, M. I. & Nelmes, R. J. (2006). *Chem. Soc. Rev.* **35**, 943–963. 10.1039/b517777b 17003900

[bb21] McMahon, M. I., Nelmes, R. J. & Rekhi, S. (2001). *Phys. Rev. Lett.* **87**, 255502. 10.1103/PhysRevLett.87.255502 11736587

[bb22] McMahon, M. I., Nelmes, R. J., Schwarz, U. & Syassen, K. (2006). *Phys. Rev. B*, **74**, 140102.

[bb23] McMahon, M. I., Rekhi, S. & Nelmes, R. J. (2001). *Phys. Rev. Lett.* **87**, 055501. 10.1103/PhysRevLett.87.055501 11497781

[bb24] Merlini, M. & Hanfland, M. (2013). *High. Press. Res.* **33**, 511–522.

[bb25] Nelmes, R. J., Allan, D. R., McMahon, M. I. & Belmonte, S. A. (1999). *Phys. Rev. Lett.* **83**, 4081–4084.

[bb26] Nelmes, R. J., McMahon, M. I., Loveday, J. S. & Rekhi, S. (2002). *Phys. Rev. Lett.* **88**, 155503. 10.1103/PhysRevLett.88.155503 11955205

[bb27] Perez-Mato, J. M., Elcoro, L., Aroyo, M. I., Katzke, H., Tolédano, P. & Izaola, Z. (2006). *Phys. Rev. Lett.* **97**, 115501. 10.1103/PhysRevLett.97.115501 17025897

[bb28] Petříček, V., Dušek, M. & Palatinus, L. (2014). *Z. Kristallogr.* **229**, 345–352.

[bb1] Rigaku Oxford Diffraction (2014). *CrysAlis PRO* software system, Version 1.171.37.35. Rigaku Oxford Diffraction, Oxford, England.

[bb29] Schwarz, U., Akselrud, L., Rosner, H., Ormeci, A., Grin, Yu. & Hanfland, M. (2003). *Phys. Rev. B*, **67**, 214101.

