# Supplementary material for: Incommensurate atomic density waves in the high-pressure IVb phase of barium
Source: IUCrJ. 2017 Jan 21;4(Pt 2):152–7. doi: 10.1107/S2052252517000264 (PMC5330526; doi:10.1107/S2052252517000264)
Supplement: Supplementary file 8 [file m-04-00152-sup8.pdf]

IUCrJ

Volume 4 (2017)

Supporting information for article:

Incommensurate atomic density waves in the high-pressure IVb phase of barium

Alla Arakcheeva, Maxim Bykov, Elena Bykova, Leonid Dubrovinsky, Phil Pattison, Vladimir Dmitriev and Gervais Chapuis

S1. Selection between the composite (COMP) and the incommensurately modulated structure (IM) models

S1.1. Preliminary analysis

X-ray diffraction experiments and their preliminary analyses are independent of any structure model. For each pressure, each reflection was characterized by its experimental intensity, standard uncertainties and indices, *hklm*, according to the wave vector $\mathbf{H} = h\mathbf{a^*} + k\mathbf{b^*} + l\mathbf{c^*} + m\mathbf{q}$, where the reciprocal lattice vectors **a***, **b*** and **c*** correspond to the unit cell parameters $a \approx b \approx 11.5$ and $c \approx 4.6$ Å; $\mathbf{q} = \beta\mathbf{b^*} + \gamma\mathbf{c^*} \approx 0.1\mathbf{b^*} + 1.36\mathbf{c^*}$. The unit cell parameters were refined along with β and γ over all the reflections of the data collection at each pressure.

S1.2. Description of the experimental data using the COMP and IM structure models

The analysis of the reflection distribution in reciprocal space allows two different interpretations of the experimental data.

Incommensurately modulated model (IM). The experimental reflections fit the monoclinic superspace group $P2_1/b(0\beta\gamma)00$ with the indicated unit cell parameters. *hkl*0 reflections are the main ones whereas *hklm (m≠0)* reflections are satellites.

Composite model (COMP). In the frame of the superspace group $P2_1/b(0\beta\gamma)00$, the reflections are considered to originate from two parts, *the host* and *the guest* substructures. The superspace group and lattice parameters of the *host* are identical to those in the IM model. The matrix (1000 / 0100 / 0001 / 0010) transforms the *guest* lattice to the *host*. Hence, the guest lattice has the same *a* and *b*, but has its own lattice parameter $c_G \approx 3.4$ Å and the monoclinic angle $\alpha_G \approx 91°$. Reflections *hkl*0 and *hk*0*m* are the main ones for the *host* and *guest* parts, respectively. Reflections with $l \neq 0$ and $m \neq 0$ are satellites. If the intensities of the satellites are negligible or zero, then both composite parts are periodic; aperiodicity appears from the irrational value of c_H/c_G.

Two different interpretations of the experimental data mainly concern the reflections in terms of "main" and "satellite".

For example, in IM, reflections *hk01, hk02 and hk03* are considered as satellites of the *1st, 2nd and 3rd* order, respectively, while they are main reflections in COMP. A distribution of the reflections with $I > 1.5\sigma(I)$ is presented in Table S1 for two models, COMP and IM, at room temperature and different pressures.

S1.3. Similarity and difference between structure models COMP and IM

Both IM and COMP models of BaIV-b have been tested and refined for each of the six different pressures. In both models, four sites of Ba atoms describe the structure framework, which is called *host* in COMP. In both models, these sites correspond to the unit cell with c ≈ 4.6 Å. Similar average

atomic coordinates and very weak displacive modulations from their positions characterize them. In both models, only one site describes Ba atoms located in the channels formed by the framework. These atoms form a chain in each channel. The chains are called *guest* in COMP. In COMP, the chain of atoms are slightly modulated along the channels with the average distance Ba - Ba = $c_G \approx 3.4$ Å. On the other hand, a strong displacive modulation along the channel with the average distance Ba - Ba = 4.6 Å is observed in the IM model.

S1.4. Reliability indices confirm the IM model

Details of the model refinements are listed in Supporting information (Tables 2 - 7) for both COMP and IM models at six different pressures. The quality of the experimental data is not identical for all pressures. This fact affects the absolute value of reliability indices. However, reliability indexes calculated for all main and satellite reflections are systematically lower for the IM model at all pressures. This justifies the high preference of IM in comparison to COMP. The comparison of R_{all}, R_{main}, R_{1st} and R_{2nd} are shown in Figure S1.

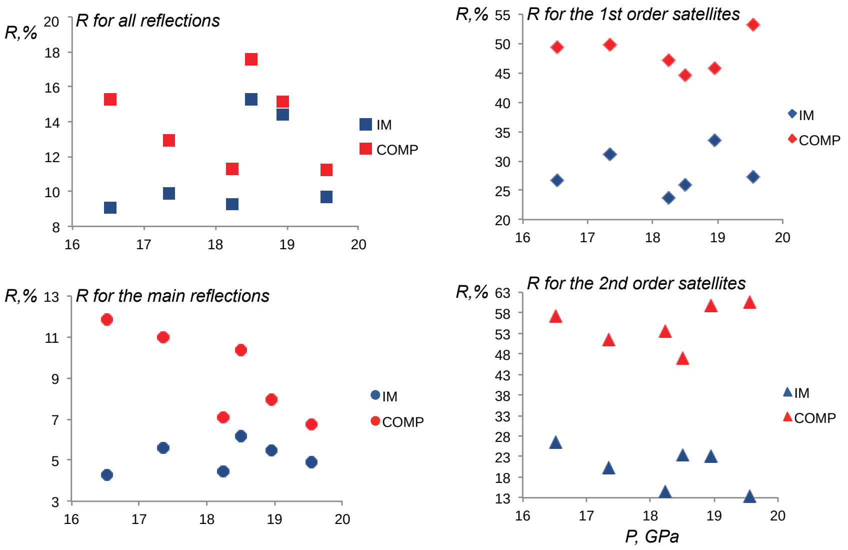

Figure S1 Comparison of R_{all}, R_{main}, R_{1st} and R_{2nd} for COMP (red) and IM (blue) models.

Table S1 BaIV-b at 16.5 - 21.8 GPa. Distribution of reflections with $I > 1.5\sigma(I)$ in two models, COMP and IM, at room temperature and different pressures

	16.5 GPa		17.4 GPa		18.2 GPa		18.5 GPa		19.0 GPa		19.6 GPa	
All reflections	438		585		655		875		548		661	
Model:	IM	COMP	IM	COMP	IM	COMP	IM	COMP	IM	COMP	IM	COMP
Main, %; type of reflections	164, 37%; *hkl*0	277, 63%; *hkl*0, *hk*0*m*	230, 39%; *hkl*0	421, 72%; *hkl*0, *hk*0*m*	195, 30%; *hkl*0	403, 62%; *hkl*0, *hk*0*m*	237, 27%; *hkl*0	476, 54%; *hkl*0, *hk*0*m*	124, 23%; *hkl*0	246. 45%; *hkl*0, *hk*0*m*	196, 30%; *hkl*0	359, 54%; *hkl*0, *hk*0*m*
1st order satellites; type of reflections	151; *hkl*1	121; *hk*11 and *hkl*1, *l* > 3	220; *hkl*1	139; *hk*11 and *hkl*1, *l* > 3	283; *hkl*1	216; *hk*11 and *hkl*1, *l* > 3	373; *hkl*1	306; *hk*11 and *hkl*1, *l* > 3	237; *hkl*1	235; *hk*11 and *hkl*1, *l* > 3	262; *hkl*1	244; *hk*11 and *hkl*1, *l* > 3
2nd order satellites; type of reflections	68; *hkl*2	19; *hk*22 and *hkl*2, *l* > 3	77; *hkl*2	16; *hk*22 and *hkl*2, *l* > 3	123; *hkl*2	33; *hk*22 and *hkl*2, *l* > 3	176; *hkl*2	76; *hk*22 and *hkl*2, *l* > 3	106; *hkl*2	53; *hk*22 and *hkl*2, *l* > 3	115; *hkl*2	41; *hk*22 and *hkl*2, *l* > 3
3rd order satellites; type of reflections	55; *hkl*3	21; *hk*33 and *hkl*3, *l* > 3	58; *hkl*3	9; *hk*33 and *hkl*3, *l* > 3	54; *hkl*3	3; *hk*33 and *hkl*3, *l* > 3	89; *hkl*3	17; *hk*33 and *hkl*3, *l* > 3	81; *hkl*3	14; *hk*33 and *hkl*3, *l* > 3	88; *hkl*3	17; *hk*33 and *hkl*3, *l* > 3

S1.5. Details of the structure refinements of BaIV-b at different pressures using two models, composite (COMP) and incommensurately modulated (MI)

Table S2 Crystallographic characteristics and details of the structure refinements of BaIV-b at **19.6** GPa

	Composite model		Incommensurately modulated model
	Host	Guest	
Unit cell parameters	*a* = 11.439 Å	*a* = 11.439 Å	*a* = 11.439 Å
	b = 11.530 Å	*b* = 11.530 Å	*b* = 11.530 Å
	c = 4.594 Å	*c* = 3.375 Å	*c* = 4.594 Å
	α = 90° β = 90°	α = 91.794°	α = 90°
		β = 90°	β = 90°
	γ = 90°	γ = 90°	γ = 90°
	V = 606 $Å^3$	V = 445 $Å^3$	V = 606 $Å^3$
W-matrix	W1 = 1000/0100/0010/0001	W2 = 1000/0100/0001/0010	-
Superspace group	$P2_1$/b(0βγ)00	$P2_1$/b(0βγ)00	$P2_1$/b(0βγ)00
**q**-vector	0.107**b*** + 1.361**c***	-0.0786**b*** + 0.735**c***	0.107**b*** + 1.361**c***
No. of reflections:			
Measured; $I_{av} > 2\sigma(I)$	15944; 643		15944; 643
No.of refl. / No. of parameters	8.8		8.8
Reliability indices R (Rw),% :			
All refl.	11.20 (7.98)		9.66 (6.73)
Main refl.	6.73 (6.11)		4.93 (4.57)
1st order satellites	53.16 (62.14)		27.20 (23.55)
2nd order satellites	60.67 (66.80)		13.41 (12.45)
3rd order satellites	44.97 (50.35)		15.76 (15.82)

Table S3 Crystallographic characteristics and details of the structure refinements of BaIV-b at **19.0** GPa

	Composite model		Incommensurately modulated model
	Host	Guest	
Unit cell parameters	*a* = 11.500 Å	*a* = 11.546 Å	*a* = 11.500 Å
	b = 11.553 Å	*b* = 11.588 Å	*b* = 11.553 Å
	c = 4.6035 Å	*c* = 3.390 Å	*c* = 4.6035 Å
	α = 90°	α = 92.05°	α = 90°
	β = 90°	β = 90°	β = 90°
	γ = 90°	γ = 90°	γ = 90°
	V = 612 $Å^3$	V = 450 $Å^3$	V = 612 $Å^3$
W-matrix	W1 = 1000/0100/0010/0001	W2 = 1000/0100/0001/0010	-
Superspace group	$P2_1$/b(0βγ)00	$P2_1$/b(0βγ)00	$P2_1$/b(0βγ)00
**q**-vector	0.122**b*** + 1.358**c***	-0.0898**b*** + 0.736**c***	0.122**b*** + 1.358**c***
No. of reflections:			
Measured; $I_{av} > 2\sigma(I)$	18445; 515		18445; 515
No.of refl. / No. of parameters	7.5		7.5
Reliability indices R (Rw),% :			
All refl.	15.18 (9.78)		14.41 (9.63)
Main refl.	7.94 (6.60)		5.46 (5.13)
1st order satellites	45.83 (49.53)		33.59 (29.54)
2nd order satellites	59.95 (73.51)		23.25 (22.19)
3rd order satellites	52.06 (54.03)		22.40 (24.45)

Table S4 Crystallographic characteristics and details of the structure refinements of BaIV-b at **18.5** GPa

	Composite model		Incommensurately modulated model
	Host	Guest	
Unit cell parameters	*a* = 11.546 Å	*a* = 11.546 Å	*a* = 11.546 Å
	b = 11.588 Å	*b* = 11.588 Å	*b* = 11.588 Å
	c = 4.599 Å	*c* = 3.394 Å	*c* = 4.599 Å
	α = 90°	α = 92.08°	α = 90°
	β = 90°	β = 90°	β = 90°
	γ = 90°	γ = 90°	γ = 90°
	V = 615 $Å^3$	V = 454 $Å^3$	V = 615 $Å^3$
W-matrix	W1 = 1000/0100/0010/0001	W2 = 1000/0100/0001/0010	-
Superspace group	$P2_1$/b(0βγ)00	$P2_1$/b(0βγ)00	$P2_1$/b(0βγ)00
**q**-vector	0.124**b*** + 1.355**c***	-0.0915**b*** + 0.73801**c***	0.124**b*** + 1.355**c***
No. of reflections:			
Measured; $I_{av} > 2\sigma(I)$	15553; 714		15553; 714
No.of refl. / No. of parameters	10.3		10.3
Reliability indices R (Rw),% :			
All refl.	17.59 (12.29)		15.31 (10.46)
Main refl.	10.35 (8.53)		6.15 (5.41)
1st order satellites	44.66 (49.07)		25.90 (24.13)
2nd order satellites	47.06 (52.85)		23.42 (22.60)
3rd order satellites	42.42 (38.30)		25.08 (27.08)

Table S5 Crystallographic characteristics and details of the structure refinements of BaIV-b at **18.2** GPa

	Composite model		Incommensurately modulated model
	Host	Guest	
Unit cell parameters	*a* = 11.531 Å	*a* = 11.531 Å	*a* = 11.531 Å
	b = 11.653Å	*b* = 11.653Å	*b* = 11.653Å
	c = 4.6109 Å	*c* = 3.376 Å	*c* = 4.6109 Å
	α = 90°	α = 91.53°	α = 90°
	β = 90°	β = 90°	β = 90°
	γ = 90°	γ = 90°	γ = 90°
	V = 620 $Å^3$	V = 454 $Å^3$	V = 620 $Å^3$
W-matrix	W1 = 1000/0100/0010/0001	W2 = 1000/0100/0001/0010	-
Superspace group	$P2_1$/b(0βγ)00	$P2_1$/b(0βγ)00	$P2_1$/b(0βγ)00
**q**-vector	0.092**b*** + 1.366**c***	-0.06735**b*** + 0.73206**c***	0.092**b*** + 1.366**c***
No. of reflections:			
Measured; I_{av}> 2σ(*I*)	18573; 629		18573; 629
No.of refl. / No. of parameters	9.0		9.0
Reliability indices R (Rw),% :			
All refl.	11.33 (7.64)		9.28 (6.58)
Main refl.	7.07 (5.77)		4.45 (4.33)
1st order satellites	47.21 (54.80)		23.75 (20.08)
2nd order satellites	53.48 (64.41)		14.40 (13.95)
3rd order satellites	61.11 (71.39)		29.10 (32.27)

Table S6 Crystallographic characteristics and details of the structure refinements of BaIV-b at **17.4** GPa

	Composite model		Incommensurately modulated model
	Host	Guest	
Unit cell parameters	*a* = 11.615 Å	*a* = 11.615 Å	*a* = 11.615 Å
	b = 11.626Å	*b* = 11.626Å	*b* = 11.626Å
	c = 4.634 Å	*c* = 3.388 Å	*c* = 4.634 Å
	α = 90°	α = 91.04°	α = 90°
	β = 90°	β = 90°	β = 90°
	γ = 90°	γ = 90°	γ = 90°
	V = 626 $Å^3$	V = 457 $Å^3$	V = 626 $Å^3$
W-matrix	W1 = 1000/0100/0010/0001	W2 = 1000/0100/0001/0010	-
Superspace group	$P2_1$/b(0βγ)00	$P2_1$/b(0βγ)00	$P2_1$/b(0βγ)00
**q**-vector	0.062**b*** + 1.368**c***	-0.06735**b*** + 0.73206**c***	0.062**b*** + 1.368**c***
No. of reflections:			
Measured; $I_{av} > 2\sigma(I)$	18897; 557		18897; 557
No.of refl. / No. of parameters	7.96		7.96
Reliability indices R (Rw),% :			
All refl.	12.92 (9.68)		9.90 (7.97)
Main refl.	10.99 (9.11)		5.59 (5.96)
1st order satellites	49.75 (57.57)		31.16 (31.41)
2nd order satellites	51.56 (66.62)		20.22 (21.83)
3rd order satellites	40.12 (39.07)		34.34 (37.94)

Table S7 Crystallographic characteristics and details of the structure refinements of BaIV-b at **16.5** GPa

	Composite model		Incommensurately modulated model
	Host	Guest	
Unit cell parameters	*a* = 11.608 Å	*a* = 11.615 Å	*a* = 11.608 Å
	b = 11.634Å	*b* = 11.626Å	*b* = 11.634Å
	c = 4.634 Å	*c* = 3.405 Å	*c* = 4.634 Å
	α = 90°	α = 91.02°	α = 90°
	β = 90°	β = 90°	β = 90°
	γ = 90°	γ = 90°	γ = 90°
	V = 626 $Å^3$	V = 460 $Å^3$	V = 626 $Å^3$
W-matrix	W1 = 1000/0100/0010/0001	W2 = 1000/0100/0001/0010	-
Superspace group	$P2_1$/b(0βγ)00	$P2_1$/b(0βγ)00	$P2_1$/b(0βγ)00
**q**-vector	0.061**b*** + 1.361**c***	-0.0448**b*** + 0.73475**c***	0.061**b*** + 1.361**c***
No. of reflections:			
Measured; $I_{av} > 2σ(I)$	20460; 399		20460; 399
No.of refl. / No. of parameters	5.7		5.7
Reliability indices R (Rw),% :			
All refl.	15.30 (11.13)		9.09 (6.38)
Main refl.	11.83 (9.87)		4.30 (4.12)
1st order satellites	49.39 (55.80)		26.72 (24.25)
2nd order satellites	57.35 (79.74)		26.65 (28.33)
3rd order satellites	46.76 (50.05)		49.14 (49.11)

S2. Structural information for the refined IM model of BaIV-b phase

Four atomic sites, Ba1, Ba2, Ba3 and Ba4 (Tables 8 - 13) are needed to define the framework in the monoclinic (3+1)D superspace group $P2_1/b(0\beta\gamma)00$. These four sites are strongly (>99%) correlated among each other, so that only two of them are independent. The correlations appear owing to the pseudo-tetragonal symmetry of the framework. The following constrains have been applied to avoid the correlations:

Ba1 and Ba2 are linked by the local operator (*x*1, 0.5-*x*2, *x*3, *x*4);

Ba3 and Ba4 are linked by the local operator (*x*1+0.5, *x*2+0.5, *x*3, *x*4).

Atomic parameters refined for BaIV-b at different pressure are listed in Table 8 - 13.

Table S8 **BaIV-b at 19.6 GPa.** Final coordinates, isotropic displacement parameters and Fourier amplitudes of the displacive modulation function. The waves are sorted by the term s for sine, c for cosines and order n.

Atom	Wave	x	y	z	Uiso
Ba1		0.0008(8)	0.10107(18)	0.2505	0.0217(12)
	s,1	-0.0043(4)	-0.0003(15)	0.0004(6)	
	c,1	-0.0021(15)	-0.0003(5)	0.0033(17)	
	s,2	-0.0005(6)	-0.0031(19)	0.0026(9)	
	c,2	-0.0016(14)	0.0008(7)	-0.0297(14)	
	s,3	0.0012(17)	0.000(3)	-0.0005(8)	
	c,3	0.0110(16)	0.0005(9)	-0.010(2)	
Ba2		0.0008(8)	0.39893(18)	0.2505	0.0217(12)
	s,1	-0.0043(4)	0.0003(15)	0.0004(6)	
	c,1	-0.0021(15)	0.0003(5)	0.0033(17)	
	s,2	-0.0005(6)	0.0031(19)	0.0026(9)	
	c,2	-0.0016(14)	-0.0008(7)	-0.0297(14)	
	s,3	0.0012(17)	0.000(3)	-0.0005(8)	
	c,3	0.0110(16)	-0.0005(9)	-0.010(2)	
Ba3		0.6489(2)	0.2544(16)	0.2507	0.0278(18)

	s,1	0.0016(14)	0.0003(5)	0.0004(8)	
	c,1	-0.0011(6)	0.004(2)	-0.004(3)	
	s,2	0.0071(19)	0.0005(10)	0.0015(12)	
	c,2	-0.0003(8)	-0.005(2)	0.002(4)	
	s,3	-0.0023(19)	-0.0005(7)	-0.0033(9)	
	c,3	-0.0016(13)	0.000(2)	-0.006(5)	
Ba4		1.1489(2)	0.7544(16)	0.2507	0.0278(18)
	s,1	0.0016(14)	0.0003(5)	0.0004(8)	
	c,1	-0.0011(6)	0.004(2)	-0.004(3)	
	s,2	0.0071(19)	0.0005(10)	0.0015(12)	
	c,2	-0.0003(8)	-0.005(2)	0.002(4)	
	s,3	-0.0023(19)	-0.0005(7)	-0.0033(9)	
	c,3	-0.0016(13)	0.000(2)	-0.006(5)	
Ba1g		0.2539(13)	0.0049(10)	0.144(2)	0.0120(17)
	s,1	-0.007(2)	0.003(2)	0.129(2)	
	c,1	0.0015(19)	0.0163(16)	0.193(2)	
	s,2	-0.001(3)	-0.012(2)	-0.049(2)	
	c,2	-0.0010(14)	0.0044(17)	0.109(2)	
	s,3	-0.0123(18)	0.0025(15)	-0.093(2)	
	c,3	-0.006(2)	-0.009(2)	0.041(3)	

Ba1g corresponds to channel atoms

Table S9 **BaIV-b at 19.0 GPa.** Final coordinates, isotropic displacement parameters and Fourier amplitudes of the displacive modulation function. The waves are sorted by the term s for sine, c for cosines and order n.

Atom	Wave	x	y	z	Uiso
Ba1		0.0053(8)	0.1016(4)	0.2515(16)	0.0188(16)
	s,1	-0.0041(5)	0.005(2)	-0.0020(7)	
	c,1	-0.0050(15)	-0.0013(7)	-0.007(3)	
	s,2	-0.0011(8)	0.003(2)	0.0026(11)	
	c,2	0.0069(16)	0.0024(9)	-0.007(2)	
	s,3	0.0031(14)	-0.0207(13)	-0.0039(11)	
	c,3	0.007(2)	-0.0004(11)	-0.019(5)	
Ba2		0.0053(8)	0.3984(4)	0.2515(16)	0.0188(16)
	s,1	-0.0041(5)	-0.005(2)	-0.0020(7)	
	c,1	-0.0050(15)	0.0013(7)	-0.007(3)	
	s,2	-0.0011(8)	-0.003(2)	0.0026(11)	
	c,2	0.0069(16)	-0.0024(9)	-0.007(2)	
	s,3	0.0031(14)	0.0207(13)	-0.0039(11)	
	c,3	0.007(2)	0.0004(11)	-0.019(5)	
Ba3		0.6481(3)	0.2458(14)	0.247(2)	0.0230(17)
	s,1	0.005(2)	0.0018(7)	0.0006(9)	
	c,1	-0.0005(6)	-0.0047(17)	0.002(4)	
	s,2	0.000(2)	0.0018(10)	-0.0004(12)	
	c,2	0.0006(9)	-0.009(3)	0.014(5)	
	s,3	0.007(2)	-0.0001(12)	-0.0042(11)	
	c,3	0.0015(10)	0.017(2)	0.009(4)	
Ba4		1.1481(3)	0.7458(14)	0.247(2)	0.0230(17)
	s,1	0.005(2)	0.0018(7)	0.0006(9)	
	c,1	-0.0005(6)	-0.0047(17)	0.002(4)	
	s,2	0.000(2)	0.0018(10)	-0.0004(12)	

	c,2	0.0006(9)	-0.009(3)	0.014(5)	
	s,3	0.007(2)	-0.0001(12)	-0.0042(11)	
	c,3	0.0015(10)	0.017(2)	0.009(4)	
Ba1g		0.2443(14)	0.0001(7)	0.136(2)	0.0134(18)
	s,1	-0.00558(18)	0.0054(18)	0.139(3)	
	c,1	0.0033(18)	0.0051(18)	0.201(3)	
	s,2	-0.0043(18)	-0.0052(18)	-0.051(3)	
	c,2	0.0041(18)	0.0085(18)	0.125(2)	
	s,3	-0.0044(18)	0.0051(18)	-0.089(2)	
	c,3	-0.0037(18)	-0.0077(18)	0.035(3)	

Ba1g corresponds to channel atoms

Table S10 **BaIV-b at 18.5 GPa.** Final coordinates, isotropic displacement parameters and Fourier amplitudes of the displacive modulation function. The waves are sorted by the term s for sine, c for cosines and order n.

Atom	Wave	x	y	z	Uiso
Ba1		-0.0003(4)	0.1011(2)	0.2553(12)	0.0099(13)
	s,1	-0.0044(3)	0.0172(6)	-0.0034(6)	
	c,1	-0.0030(6)	-0.0009(5)	0.0135(18)	
	s,2	-0.0022(4)	-0.0001(6)	0.0014(8)	
	c,2	0.0051(11)	0.0066(7)	0.013(2)	
	s,3	-0.0153(8)	0.0071(8)	-0.0044(8)	
	c,3	-0.0036(10)	0.0038(7)	-0.019(3)	
Ba2		-0.0003(4)	0.3989(2)	0.2553(12)	0.0099(13)
	s,1	-0.0044(3)	-0.0172(6)	-0.0034(6)	
	c,1	-0.0030(6)	0.0009(5)	0.0135(18)	
	s,2	-0.0022(4)	0.0001(6)	0.0014(8)	
	c,2	0.0051(11)	-0.0066(7)	0.013(2)	
	s,3	-0.0153(8)	-0.0071(8)	-0.0044(8)	
	c,3	-0.0036(10)	-0.0038(7)	-0.019(3)	
Ba3		0.64871(19)	0.2413(8)	0.248(2)	0.0203(12)
	s,1	0.0024(10)	-0.0013(5)	-0.0005(7)	
	c,1	-0.0005(4)	0.0063(13)	-0.004(4)	
	s,2	-0.0006(11)	0.0005(7)	0.0009(10)	
	c,2	-0.0012(6)	0.0064(12)	0.001(2)	
	s,3	0.0097(11)	-0.0030(9)	-0.0038(8)	
	c,3	0.0002(6)	0.0042(14)	-0.009(4)	
Ba4		1.14871(19)	0.7413(8)	0.248(2)	0.0203(12)
	s,1	0.0024(10)	-0.0013(5)	-0.0005(7)	
	c,1	-0.0005(4)	0.0063(13)	-0.004(4)	
	s,2	-0.0006(11)	0.0005(7)	0.0009(10)	

	c,2	-0.0012(6)	0.0064(12)	0.001(2)	
	s,3	0.0097(11)	-0.0030(9)	-0.0038(8)	
	c,3	0.0002(6)	0.0042(14)	-0.009(4)	
Ba1g		0.2538(11)	0.0006(6)	0.1354(17)	0.0073(13)
	s,1	-0.0008(18)	0.0111(10)	0.1376(18)	
	c,1	-0.0015(14)	0.0135(10)	0.207(2)	
	s,2	0.0080(19)	-0.0051(12)	-0.057(2)	
	c,2	0.0020(10)	0.0054(12)	0.1249(18)	
	s,3	-0.0030(13)	0.0069(8)	-0.0965(16)	
	c,3	-0.0002(17)	-0.0020(14)	0.034(3)	

Ba1g corresponds to channel atoms

Table S11 **BaIV-b at 18.2 GPa.** Final coordinates, isotropic displacement parameters and Fourier amplitudes of the displacive modulation function. The waves are sorted by the term s for sine, c for cosines and order n.

Atom	Wave	x	y	z	Uiso
Ba1		-0.0029(13)	0.10109(19)	0.2545(14)	0.0173(8)
	s,1	-0.0034(3)	0.0018(15)	-0.0002(8)	
	c,1	-0.0018(17)	0.0000(4)	0.009(2)	
	s,2	-0.0001(5)	0.0008(15)	0.0027(11)	
	c,2	-0.0010(12)	-0.0002(7)	0.000(3)	
	s,3	0.005(3)	-0.0131(8)	-0.0012(10)	
	c,3	0.009(2)	0.0001(9)	-0.001(3)	
Ba2		-0.0029(13)	0.39891(19)	0.2545(14)	0.0173(8)
	s,1	-0.0034(3)	-0.0018(15)	-0.0002(8)	
	c,1	-0.0018(17)	0.0000(4)	0.009(2)	
	s,2	-0.0001(5)	-0.0008(15)	0.0027(11)	
	c,2	-0.0010(12)	0.0002(7)	0.000(3)	
	s,3	0.005(3)	0.0131(8)	-0.0012(10)	
	c,3	0.009(2)	-0.0001(9)	-0.001(3)	
Ba3		0.64874(15)	0.2524(16)	0.2503(14)	0.0203(8)
	s,1	0.0003(17)	-0.0014(8)	0.0010(8)	
	c,1	-0.0002(4)	0.001(2)	-0.002(2)	
	s,2	0.000(2)	0.0001(7)	-0.0003(10)	
	c,2	-0.0008(6)	-0.0001(15)	-0.005(3)	
	s,3	0.0040(17)	0.0012(9)	-0.0045(10)	
	c,3	0.0003(9)	0.0157(10)	-0.009(2)	
Ba4		1.14874(15)	0.7524(16)	0.2503(14)	0.0203(8)
	s,1	0.0003(17)	-0.0014(8)	0.0010(8)	
	c,1	-0.0002(4)	0.001(2)	-0.002(2)	
	s,2	0.000(2)	0.0001(7)	-0.0003(10)	

	c,2	-0.0008(6)	-0.0001(15)	-0.005(3)	
	s,3	0.0040(17)	0.0012(9)	-0.0045(10)	
	c,3	0.0003(9)	0.0157(10)	-0.009(2)	
Ba1g		0.2454(11)	-0.0002(11)	0.135(3)	0.0125(16)
	s,1	0.008(3)	0.015(2)	0.146(3)	
	c,1	0.0008(17)	0.015(2)	0.180(3)	
	s,2	0.012(2)	0.007(3)	-0.045(4)	
	c,2	0.0015(15)	0.0152(13)	0.114(2)	
	s,3	0.0054(19)	0.0123(14)	-0.093(2)	
	c,3	0.0081(18)	0.003(2)	0.035(3)	

Ba1g corresponds to channel atoms

Table S12 **BaIV-b at 17.4 GPa.** Final coordinates, isotropic displacement parameters and Fourier amplitudes of the displacive modulation function. The waves are sorted by the term s for sine, c for cosines and order n.

Atom	Wave	x	y	z	Uiso
Ba1		0.0025(8)	0.1014(2)	0.2502	0.0115(11)
	s,1	0.0005(4)	-0.0075(15)	-0.0006(13)	
	c,1	-0.0043(11)	-0.0001(8)	-0.007(3)	
	s,2	-0.0017(8)	0.0131(16)	-0.006(2)	
	c,2	0.0001(13)	-0.0014(15)	0.014(2)	
	s,3	0.0019(19)	0.007(2)	-0.0004(15)	
	c,3	-0.0145(8)	-0.0029(9)	0.006(5)	
Ba2		0.0025(8)	0.3986(2)	0.2502	0.0115(11)
	s,1	0.0005(4)	0.0075(15)	-0.0006(13)	
	c,1	-0.0043(11)	0.0001(8)	-0.007(3)	
	s,2	-0.0017(8)	-0.0131(16)	-0.006(2)	
	c,2	0.0001(13)	0.0014(15)	0.014(2)	
	s,3	0.0019(19)	-0.007(2)	-0.0004(15)	
	c,3	-0.0145(8)	0.0029(9)	0.006(5)	
Ba3		0.6486(2)	0.2530(18)	0.2506	0.0142(12)
	s,1	0.002(2)	0.0017(14)	-0.0008(13)	
	c,1	0.0003(6)	-0.009(3)	0.001(3)	
	s,2	0.0062(19)	-0.001(2)	0.0025(19)	
	c,2	0.0007(14)	-0.0164(17)	-0.015(3)	
	s,3	-0.008(2)	-0.0030(10)	-0.0033(16)	
	c,3	0.0012(10)	0.001(3)	0.008(6)	
Ba4		1.1486(2)	0.7530(18)	0.2506	0.0142(12)
	s,1	0.002(2)	0.0017(14)	-0.0008(13)	
	c,1	0.0003(6)	-0.009(3)	0.001(3)	
	s,2	0.0062(19)	-0.001(2)	0.0025(19)	

	c,2	0.0007(14)	-0.0164(17)	-0.015(3)	
	s,3	-0.008(2)	-0.0030(10)	-0.0033(16)	
	c,3	0.0012(10)	0.001(3)	0.008(6)	
Ba1g		0.25	-0.0169(11)	0.254(5)	0.026(2)
	s,1	0.0000(14)	-0.0037(18)	0.148(5)	
	c,1	0.0000(14)	-0.0077(18)	-0.151(3)	
	s,2	0.0000(14)	0.0054(12)	0.006(3)	
	c,2	0.0000(14)	-0.0137(15)	-0.037(6)	
	s,3	0.0054(19)	0.0123(14)	-0.093(2)	
	c,3	0.0081(18)	0.003(2)	0.035(3)	

Ba1g corresponds to channel atoms

Table S13 **BaIV-b at 16.5 GPa.** Final coordinates, isotropic displacement parameters and Fourier amplitudes of the displacive modulation function. The waves are sorted by the term s for sine, c for cosines and order n.

Atom	Wave	x	y	z	Uiso
Ba1		-0.0011(7)	0.1014(2)	0.2506	0.0174(12)
	s,1	0.0003(4)	-0.0056(16)	0.0033(11)	
	c,1	-0.0033(11)	0.0002(7)	0.008(4)	
	s,2	0.0012(8)	0.0076(16)	0.0000(12)	
	c,2	0.0011(15)	-0.0016(12)	0.018(2)	
	s,3	-0.0021(17)	-0.007(2)	-0.0031(12)	
	c,3	-0.0132(11)	0.0018(7)	-0.004(3)	
Ba2		-0.0011(7)	0.3986(2)	0.2506	0.0174(12)
	s,1	0.0003(4)	0.0056(16)	0.0033(11)	
	c,1	-0.0033(11)	-0.0002(7)	0.008(4)	
	s,2	0.0012(8)	-0.0076(16)	0.0000(12)	
	c,2	0.0011(15)	0.0016(12)	0.018(2)	
	s,3	-0.0021(17)	0.007(2)	-0.0031(12)	
	c,3	-0.0132(11)	-0.0018(7)	-0.004(3)	
Ba3		0.6488(2)	0.2532(11)	0.2504	0.0191(16)
	s,1	-0.002(2)	-0.0002(9)	-0.0011(11)	
	c,1	-0.0018(6)	-0.009(3)	0.000(3)	
	s,2	-0.0056(15)	0.0016(13)	-0.0012(15)	
	c,2	0.0012(12)	-0.011(3)	-0.005(4)	
	s,3	-0.0061(17)	0.0019(13)	0.001(3)	
	c,3	-0.0002(9)	0.0078(16)	0.022(4)	
Ba4		1.1488(2)	0.7532(11)	0.2504	0.0191(16)
	s,1	-0.002(2)	-0.0002(9)	-0.0011(11)	
	c,1	-0.0018(6)	-0.009(3)	0.000(3)	
	s,2	-0.0056(15)	0.0016(13)	-0.0012(15)	

	c,2	0.0012(12)	-0.011(3)	-0.005(4)	
	s,3	-0.0061(17)	0.0019(13)	0.001(3)	
	c,3	-0.0002(9)	0.0078(16)	0.022(4)	
Ba1g		0.25	-0.0164(11)	0.249(3)	0.036(2)
	s,1	0.0000(14)	-0.0068(16)	0.151(5)	
	c,1	0.0000(14)	-0.0136(17)	-0.153(4)	
	s,2	0.0000(14)	0.0047(12)	0.002(3)	
	c,2	0.0000(14)	-0.0091(17)	-0.046(6)	
	s,3	0.0000(14)	0.0012(15)	0.045(4)	
	c,3	0.0000(14)	0.0041(15)	0.051(4)	

Ba1g corresponds to channel atoms

S3. Details on the interatomic distance distribution

The magnitudes of the modulations are three orders smaller for all the Ba_{Fr} comparing to the magnitude of Ba_{Ch} along the *c* axis (Table S8 - S13). The magnitude of the Ba_{Ch} atom modulation is also negligible along the *a* and *b* axes (Table S8 - S13). Consequently the structure modulation essentially affects the Ba_{Ch}–Ba_{Ch} distances along the channels. In general, they vary from 2.90 to 6.91 – 7.25 Å between the nearest atoms (Fig. 4 in the main text) with an average value corresponding to the lattice parameter $c \approx 4.6$ Å. The variation of 70-85% of the Ba_{Ch}–Ba_{Ch} distances in the 2.9 - 3.8 Å range (Fig. 4, a and b in the main text) lead to their grouping in dumbbells and triplets (Fig. 4, c in the main text). The extreme values of the range are closely related to Ba-Ba distances at higher and lower pressure, respectively: 3.0 Å in the *hcp* BaV at P > 45 GPa and 3.8 Å in the *hcp* BaII at P < 12 GPa (Kinichi, 1994). More than 80% of the host atoms have Ba_{Fr}-Ba_{Fr} contacts within the narrow range of 3.2 – 3.4 Å. Hence, the appearance of the incommensurately modulated state of BaIV is directly linked to the formation of short Ba - Ba contacts in the range 2.8 - 3.0 Å. The shortest Ba_{Ch}–Ba_{Fr} contact is also approximately 2.8 Å.

The atomic density in the channels is about 4/3 smaller than what was assumed before using the composite model for BaIV-a (Kenichi, 1994). Indeed, in the Ba_{Ch}-chain, the average length of the Ba_{Ch}–Ba_{Ch} contact is about 4.6 Å given by the lattice constant *c*, while this length is about $c_G \approx 3.4$ Å in all publications. In particular, assuming a hypothetical composite model for BaIV-b, the density could be overestimated by about 7%.
